# Interferon lambda 4 variant rs12979860 is not associated with RAV NS5A Y93H in hepatitis C virus genotype 3a

**DOI:** 10.1002/hep.28533

**Published:** 2016-04-18

**Authors:** Vincent Pedergnana, David Smith, STOP‐HCV Consortium, Paul Klenerman, Eleanor Barnes, Chris C. A. Spencer, M. Azim Ansari

**Affiliations:** ^1^ Wellcome Trust Centre for Human GeneticsUniversity of OxfordOxfordUnited Kingdom; ^2^ Nuffield Department of MedicineUniversity of OxfordOxfordUnited Kingdom; ^3^ Oxford Martin SchoolUniversity of OxfordOxfordUnited Kingdom

Author names in bold designate shared co‐first authorship.

TO THE EDITOR:

Peiffer et al. recently reported^1^ an association between the host interferon lambda 4 (*INFL4*) single‐nucleotide polymorphism (SNP), rs12979860, and the NS5A resistance‐associated variant (RAV) Y93H in hepatitis C virus (HCV) genotype 1b (HCVg1b). This observation is intriguing because it directly links innate immunity to HCV viral drug resistance for the first time. A small cohort of (51) HCV genotype 3 (HCVg3) patients was included in the analysis; this subgroup analysis was underpowered and no association was observed in HCVg3. The association was also not observed in 259 patients with HCV subtype 1a.

HCVg3 infections are more difficult to treat with direct‐acting antivirals. The reason for this is unknown, but could be explained by a distinct pattern of RAVs and an increase in the prevalence of “favorable” *IFNL4* SNPs in this genotype. Here, we used a large cohort of 496 HCVg3a‐infected patients (from the BOSON clinical study^2^) and report no significant association (*P* > 0.05) between the treatment‐beneficial C/C genotype of *INFL4* and RAV site Y93H in the *NS5A* gene.

Using next‐generation sequencing,^3^ baseline viral sequences from 556 BOSON patients chronically infected with either HCVg2 or HCVg3 were obtained. The *INFL4* SNP rs12979860 was also genotyped. The cohort (total of 556) was composed of 49 (8.8%) HCVg2‐ and 507 (91.2%) HCVg3‐infected patients, of which 496 (89.2%) were subtype 3a.

Substitutions in the quasi‐species at Y93 (Y93H) were only observed in HCVg3a‐infected patients; therefore, the analysis was done on this subset of the data. The Y93H RAV was present in 11.1% (55 of 496) of the genotype 3a–infected patients; in 4.7% (23 of 496), Y93H was as the majority variant. There was no significant association between the presence of H (either as the consensus level or at any level of detection) and the beneficial C/C genotype of *INFL4* (*P* > 0.05; Table 1). Overall, our data support the Peiffer et al. hypothesis that the association between INFL4 and Y93H is specific to HCV genotype 1b.

**Table 1:** Prevalence of Y93H RAV in HCVg3a‐Infected Patients (n=496) Stratified by rs12979860 *IFNL4* SNP

	Y93H: Consensus Level n/N (%)	Y93H: All Types n/N (%)
*INFL4* C/C	12/184 (6.5)	25/184 (13.5)
*INFL4* non C/C	11/312 (3.5)	30/312 (9.6)

**Vincent Pedergnana, Ph.D.^1^**
**David Smith, B.Sc.^2^**
**STOP‐HCV Consortium**
**Paul Klenerman, F.Med.Sci.^2^**
**Eleanor Barnes, B.Sc., M.B.B.S., M.R.C.P., Ph.D.^2^**
**Chris C. A. Spencer, D.Phil.^1^**
**M. Azim Ansari, D.Phil.^2,3^**
**^1^Wellcome Trust Centre for Human Genetics**
**University of Oxford**
**Oxford, United Kingdom**
**^2^Nuffield Department of Medicine**
**University of Oxford**
**Oxford, United Kingdom**
**^3^Oxford Martin School**
**University of Oxford**
**Oxford, United Kingdom**
